# Laser Texturing to Increase the Wear Resistance of an Electrophoretic Graphene Coating on Copper Substrates

**DOI:** 10.3390/ma16155359

**Published:** 2023-07-30

**Authors:** Gabriele Baiocco, Silvio Genna, Daniel Salvi, Nadia Ucciardello

**Affiliations:** Department of Enterprise Engineering Mario Lucertini, University of Rome Tor Vergata, 00133 Rome, Italy; gabriele.baiocco@uniroma2.it (G.B.);

**Keywords:** EPD, fibre laser, Graphene, friction coefficient, surface treatments

## Abstract

In the present paper, different surface preparations are investigated with the aim of increasing the wear behaviour of an electrophoretic graphene coating on a copper plate. The study was divided into two steps: In the first step (pre-tests), to detect the most promising pretreatment technology, five different surface preparations were investigated (electropolishing, sandblasting, degreasing and pickling, laser cleaning and laser dots).In the second step, on the basis of the results of the first step, a 3^2^ full factorial plan was developed and tested; three treatment types (pickled and degreased, laser-cleaned, and laser dots) and three different voltages (30, 45 and 60 V) were adopted. Analysis of variance (ANOVA) was used to evaluate their influence on wear resistance; in particular, the maximum depth and width of the wear tracks and the coating break distance were investigated. The results of this study show that, in optimal conditions, laser treatment (particularly laser dots) canlead to as high as a four-fold increase in wear resistance.

## 1. Introduction

Over the years, copper and its alloys have played a critical role in the industrial and metallurgical sectors since properties such as ductility and conductivity ensure extensive involvement in many fields [1,2], even under wear conditions [3]. However, unsatisfactory mechanical properties such as hardness strength and wear resistance limit the application of copper and its alloys, especially under high-temperature operating conditions [4,5,6]; hence, improving wear resistance to increase the service life of copper-based components is an urgent need [3].

At present, surface treatment technologies such as thermal spraying, electroplating, laser cladding, magnetron co-sputtering, and plasma cladding are widely exploited to coat a protective layer on copper substrates and increase friction wear performance and service life [6,7,8,9,10,11,12,13,14]. Copper-based nanocomposites produced through these methods have shown a growing trend in copper surface protection due to their improved mechanical properties, leaving the physical performance of both the substrate and the matrix unaffected, thus enhancing the wear resistance through improved lubricating capability [15,16,17]. In fact, Cu composite coatings with reinforcing phases (such as ceramic or carbonaceous) benefit from both metals and fillers and demonstrate outstanding wear resistance and improved electrical and thermal conductivity and self-lubricating properties, which has led to themattracting increasing interest in industrial fields [18,19,20,21,22].

Among all of the fillers, graphene is an ideal reinforcement due to its superior properties (mechanical, electrical/thermal, and lubricating properties) and relatively inexpensive production cost [23,24,25]. Supposed state-of-the-art methods include powder metallurgy, vapour deposition, electro-spark deposition, laser cladding, and thermal spraying; however, those methods require high-temperature and high-pressure conditions [26], and the layer produced is not free from defects such as low bonding strength between the coating and the corresponding substrate. Furthermore, vapour deposition methods are complicated to manage and require demanding and costly devices that can release toxic gaseous products [27]. These critical aspects hinder the application of these strengthening methods in manufacturing Cu components for harsh service conditions; thus, a more cost-effective process of increasing copper surface performance needs to be identified [28,29]. 

Electrophoretic deposition (EPD) processes represent a valuable alternative to vapour phase deposition processes for nanostructured coatings and nanoscale films with enhanced properties [30]. In Electrophoretic deposition (EPD) processes, charged colloidal particles are dispersed in a liquid medium to migrate, under the influence of an electric field, towards oppositely charged electrodes by applying a voltage [31]. EPD is a versatile, fast, and cost-effective technique that isadjustable for specific applications due to its easy deposition rate and thickness control, which ensure coating uniformity. The advantages of EPD processes include fast deposition rates and a lack of restrictionsregarding the shape of deposition substrates, which makes the process effective in producing well-adhered coatings in a cost-effective and non-dangerous way using a simple and economical apparatus [30,32]. Also, the use of the aqueous system as the suspending medium requires lower voltages and costs, reducing the environmental impact compared to the hazardous organic liquids that are usually used [31]. Several papers have investigated the utility of the EPD techniquefor producing graphene coatings for used on copper substrates, although the majority of these studiesdiscussed the use of the EPD technique in the context of corrosion prevention [33,34,35,36,37,38,39,40]. 

Surface textures have also been proven to lower the coefficient of friction (COF); however, it is gradually modified by plastic deformation, especially with high local contact pressures [41,42]. 

A combination of surface texturing and lubricants has been applied to improve wear behaviour; the geometry printed on the surface acted as lubricant reservoirs and pits to catch the debris produced during the dry sliding conditions [43,44,45,46,47]. To the best of the authors’ knowledge, there is no work on using the EPD process and laser texturing to apply graphene over copper substrates; therefore, this work aims to fill this gap. Different types of texturing on copper substrates were developed as substrate treatments for the electrophoresis process.

Wear tests were carried out to determine the friction coefficient and the graphene coating’s durability and evaluate the laser treatment performance. The experimental results show low friction coefficients for all deposited films; however, the durability of the films was strongly influenced by the type of texturing that precedes deposition.

## 2. Materials and Methods

Samples of pure copper (99.9% wt.; 20 × 40 × 2 mm^3^) were adopted as substrates for the EPD process. The study was divided into two steps: in the first step (pre-tests), to detect the most promising pretreatment technology, five different surface preparations were investigated (electropolishing, sandblasting, pickling and degreasing, laser cleaning, and laser dots).In the second step, based on the results of the first step, a full factorial plan was developed and tested. For the sandblasting treatment (SB), the sample was blasted with aluminium oxide at 4 bars for 30 s, removing the oxide and obtaining a clean, irregular surface, and this was carried out according to previous experience [48]. The powder exploited was provided by Smyris Abrasivi S.r.l. (Pero, Mi, Italy)and characterised by a mesh of 16 with 1.2 µm average diameter and a shape factor of 0.67. The sandblasting treatment was performed at an angle of about 60° to the normal surface.

For the electropolishing (EP) pretreatment, the sample was electrochemically treated with the bath described in Table 1.The substrate was attached to the anode of a current generator, and a potential difference of 10 V was applied, resulting in an oxide-free and highly polished surface, as previously obtained in the literature [49,50]. In the degreasing and pickling (DP) treatment, the sample was first subjected to electrochemical cathodic degreasing at 8 Ampere for 3 min and subsequently immersed in a commercial degreaser (Condorine 156, Condoroil Chemical S.r.l., Casale Litta, Italy). After being rinsed in water, the sample was then dipped into a commercial chemical degreaser (744P, CondoroilChemical) for 2 min and subsequently rinsed in water.The degreasing solution was purchased from Condoroil Chemical S.r.l.

The laser texturing treatment was performed by using a pulsed 30 Wfibre source (Yb:YAG) (model YLP-RA30-1-50-20-20 by IPG Photonics, Oxford, MA, USA) equipped with a galvanometric scanning head (supplied by LASIT SpA, Naples, Italy). The laser beam was moved via two galvanometric mirrors and focused by a flat lens (F-Theta by LINOS, Göttingen, Germany) 160 mm in focal length. The laser spot at the focusing point was about 80 μm. In Table 2, the laser system characteristics are reported.

**Figure 1:**
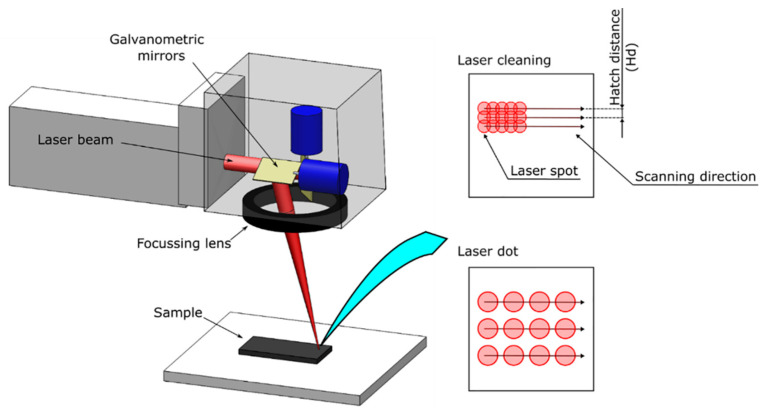
Schematic of laser treatment.

The system was controlled via a software programme that enables the management of the laser power (P), pulse frequency (F), number of repetitions (R), scanning speed (Ss), and scanning strategy. The average power can be changed by varying the power supply provided to the pumping diodes. Figure 1 depicts a schematic of the laser treatment; the scanning direction and the hatch distance (Hd), i.e., the distance between two successive scanning lines, are highlighted. By changing the pulse frequency and the scanning speed, it is possible to change the pulse overlap, i.e., the distance between two consecutive laser footprints. The laser parameters were selected based on previous experience [51,52]. The values for the two laser treatments performed—laser cleaning (LC) and laser dots (LD)— are shown in Table 3. It is worth noting that by adopting a high scanning speed (4300 mm/s), laser footprints were created without overlap. 

For the electrophoretic deposition technique, the component was placed at the anode of a DC generator (PSW250-4.5, GW Instek, New Taipei City, Taiwan) while the cathode consisted of an AISI 304 stainless steel foil. For all samples, the deposition time was set at 10 min, after which the sample was rinsed in distilled water and dried with compressed air. For the factorial plan, three different voltages were chosen: 30, 45, and 60 V. The deposition bath used consisted of a 1 g/L dispersion of GNPs (ACS Material, Pasadena, USA, whose characteristics are reported in Table 4) in distilled water. Prior to deposition, the bath was subjected to 10 min of sonication to facilitate the dispersion of the graphene using a Sonics Materials VCX 750 (Thermo Fisher Scientific Inc., Waltham, MA, USA) instrument.

In order to reduce the number of trial tests and to assess the effects of the treatments, a 3^2^ full factorial plan was developed and tested. The control factors were the treatment type and the voltage adopted during electrophoretic deposition. The control factors and their levels are reported in Table 5. As response variables, the following wear parameters were selected: the maximum depth (d [µm]) and maximum wear width (w [µm]) of the wear tracks, their combination (w × d [µm^2^]), and the coating break distance (CBD). Coating failure was declared when the coating showed a 5% increase in COF from the average plateau value. CBD was computed as the distance in which the failure happened.

After being subjected to the different pretreatments, the morphological characterisation of the samples was performed by using a 3D Surface Profiling System (Talysurf CLI 2000, Taylor Hobson, Leicester, UK) to collect 3D maps and roughness profiles. The 3D map dimensions were 1 × 1 mm^2^,with a 1 µm resolution; the Arithmetical Mean Height of the scanned area (Sa) was also collected. The roughness of the treated samples was studied while considering the most important roughness parameters: Roughness Average (Ra) and Ten Point Height of Irregularities (Rz). For each sample, 30 profiles with 12.5 mm length, an interspace of 100 µm, and a resolution of 1 µm were collected. The profiles were then elaborated through the use of surface analysis software (Talymap Universal 3.1.4), which collected Ra and Rz using a 0.8 mm Gaussian Filter.

Tribological tests were carried out using a standard tribometer (CSM Instruments, Needham, MA, USA) in the ball-on-flat configuration with a half-amplitude of 2.5 mm and a maximum speed of 5 cm/s. The tests were carried out with a normal load of 1 N using a 100Cr6 steel ball with a diameter of 6 mm. The test distance was set at 100 m. Three different samples were produced and tested for each condition to ensure the repeatability of the process. 

**Figure 2:**
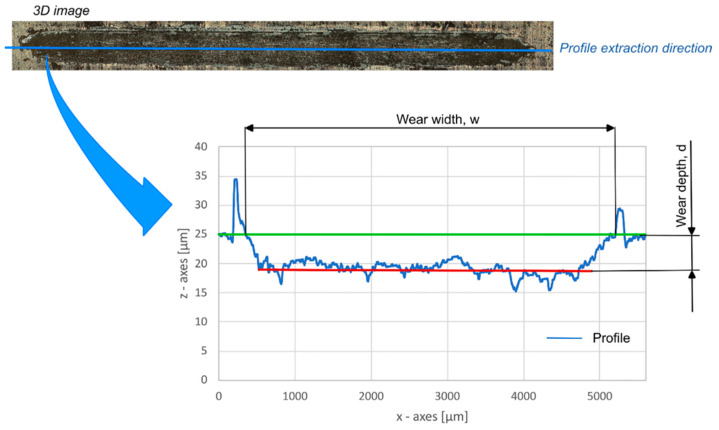
Schematic of wear track measurement: the blue line represents the acquired profile; the green one the average base profile, the red line the average lower probile.

For the samples that did not exhibit coating failure during the test, an additional 500 m test was conducted to evaluate the CBD. The maximum depth, d [µm], and the maximum wear width, w [µm], were measured via the use of a 3D digital video microscopy system (KH-8700 by Hirox, Hackensack, NJ, USA) using the MXG-2500 REZ “revolver” optics with 350× magnification and adopting the “3D tiling” function. This function allows for the system to move along 3 axes, acquiring the surface. Then, after the surface is rebuilt, the function is able to measure the profiles extracted by the 3D surface. Figure 2 depicts a schematic of wear track measurement; wear depth and the width of the tracks were calculated by extracting the profile in the middle of the tracks.

The effect of the control factors (i.e., treatment type and deposition voltage) on wear behaviour was evaluated via ANOVA. For the test, Minitab R18 software was used. The analysis was conducted at a confidence level of 95%, i.e., a control factor or a combination of more control factors is statistically significant if the *p*-value is less than 0.05. Before the analysis, our hypotheses were checked by graphically analysing the residues. The main effect plots were adopted to show the effect of a control factor on the response variables. In addition, in order to detect the effectsattributable to a combination of multiple control factors, interaction plots were generated.

## 3. Results and Discussion

### 3.1. PreliminaryTests

**Figure 3:**
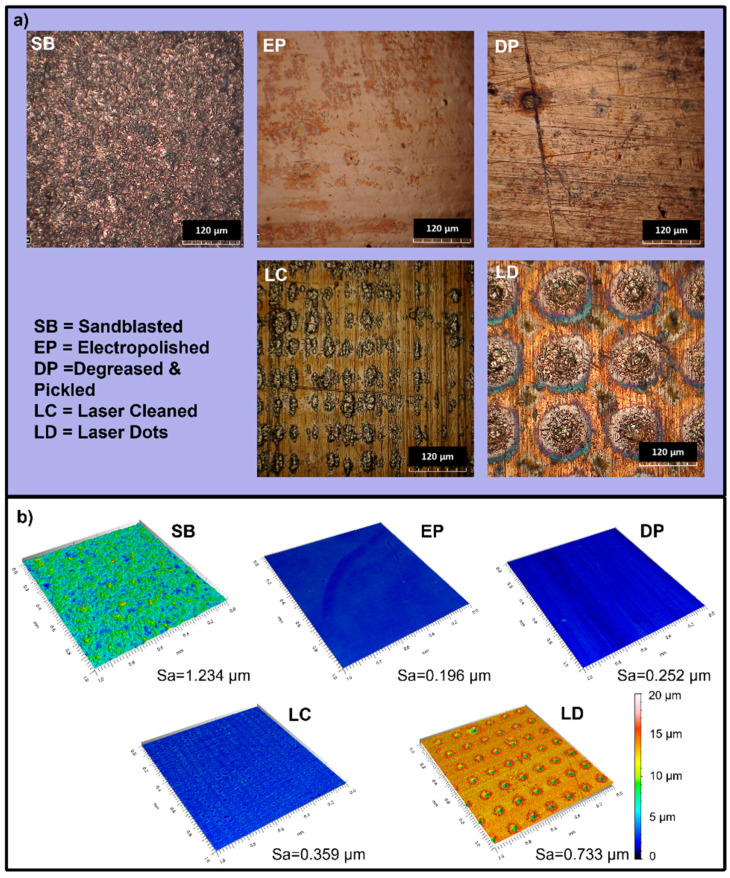
Copper substrate morphology after the pretreatment processes (**a**) optical images; (**b**) 3D surface maps and Arithmetical Mean Height (Sa) of the surfaces.

The surface pretreatment analysis was performed preliminarily by evaluating the friction coefficient of the various treated surfaces coated under the same deposition conditions (30 V). Figure 3 shows the surface morphology of the treated surfaces through optical imaging and 3D maps. The sandblasted sample shows an irregular surface due to the mechanical removal of the surface layer caused by the impacts of the abrasive powder at different angles. The EP sample and the DP sample present the most homogenous surfaces. In the DP sample, the substrate morphology is unaltered since the signs of the rolling process are still evident; on the EP surface, those signs are no longer visible. The LC surface presents an inhomogeneous morphology due to the laser’s transition. In laser cleaning, the beam surface scanning produces dots and areas with droplets of recast material. In contrast, LD treatment creates regular texturing with equally spaced dots.

It can be seen from Table 6 that the SB treatment appeared to be the roughest, which is in agreement with the images in Figure 3, with a Ra value approximately an order of magnitude higher than the other pretreatments. In contrast, the EP sample turned out to be the least rough. The laser treatments showed higher values than the DP treatment but with Ra still inferior to 1 µm.

**Figure 4:**
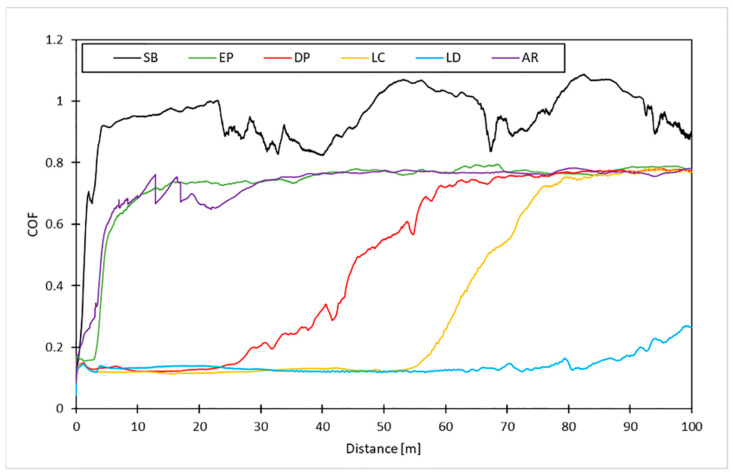
Friction curve achieved for as received samples (AR) and after EPD process at 30 V for the different surface treatments investigated.

Surface pretreatment analysis was performed preliminarily by evaluating the friction coefficient of the various treated surfaces coated with electrophoretic deposition at the same conditions; the friction curves are shown in Figure 4. 

The low friction coefficient obtained by the laser texturing and electrochemical cleaning processes, which was constant for the three preparations, indicates the presence of graphene on the surfaces. The substantial difference lies in the CBD, which is clearly higher for the LD. On the contrary, electropolishing and sandblasting exhibit extremely high friction values compared with the typical copper values from the initial metres of sliding. In addition, the high roughness typical of sandblasted surfaces made the coefficient of friction (COF) very unstable during the test. Based on these preliminary results, sandblasting and electropolishing were discarded.

### 3.2. Full Factorial Plan

**Figure 5:**
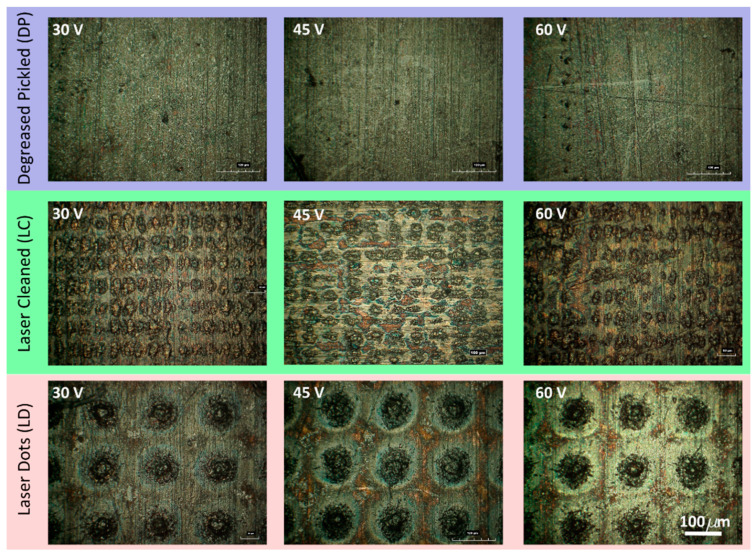
Optical images of the surface coated by EPD after different surface treatments.

Figure 5 depicts the optical images of the coatings obtained at different voltages for the three selected pretreatments. The graphene is preferentially disposed of within the dots created. In particular, in the LD surface, the graphene is arranged in the dimples’ centre; in the LC, the arrangement of graphene is inhomogeneous and follows the surface irregularities that characterise this treatment. This suggests that surface dimples promote graphene deposition. The DP treatment did not produce depressions, thereby achieving a more homogeneous deposition.

**Figure 6:**
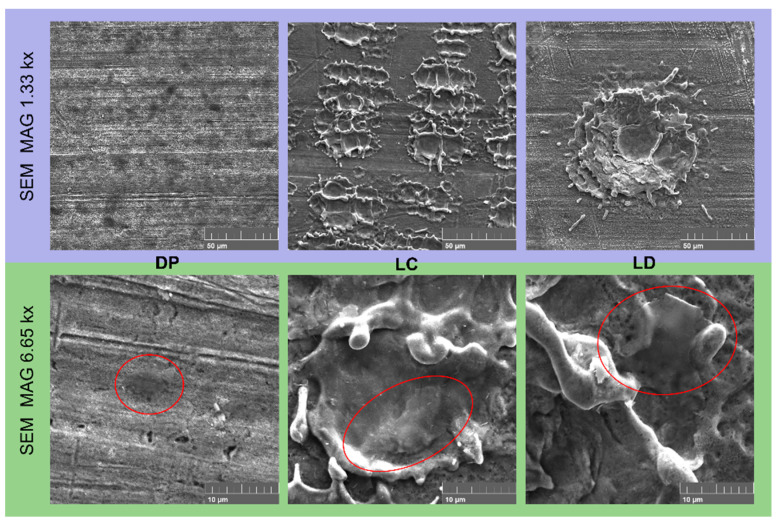
SEM images of the surface coated by EPD at 45 V after different surface treatments; examples of visible graphene platelets are circled in red.

The SEM images shown in Figure 6 reveal the presence of graphene. The laser-treated samples concentrate the graphene particles within the surface irregularities created by the treatment. In contrast, the DP samples present homogeneously distributed graphene on the surface, leading to continuous film deposition.

**Figure 7:**
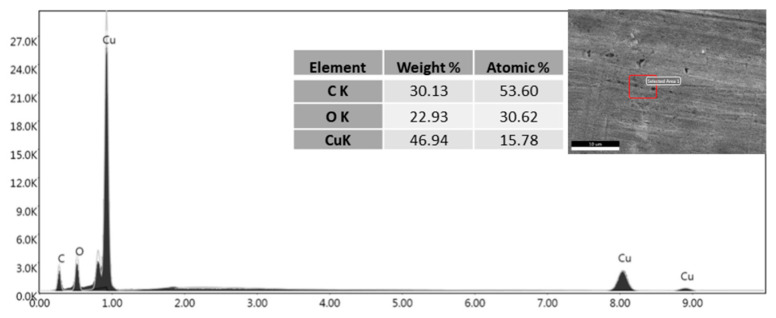
EDX analysis of the EPD coating on pickled and degreased surface coated by EPD at 45 V.

**Figure 8:**
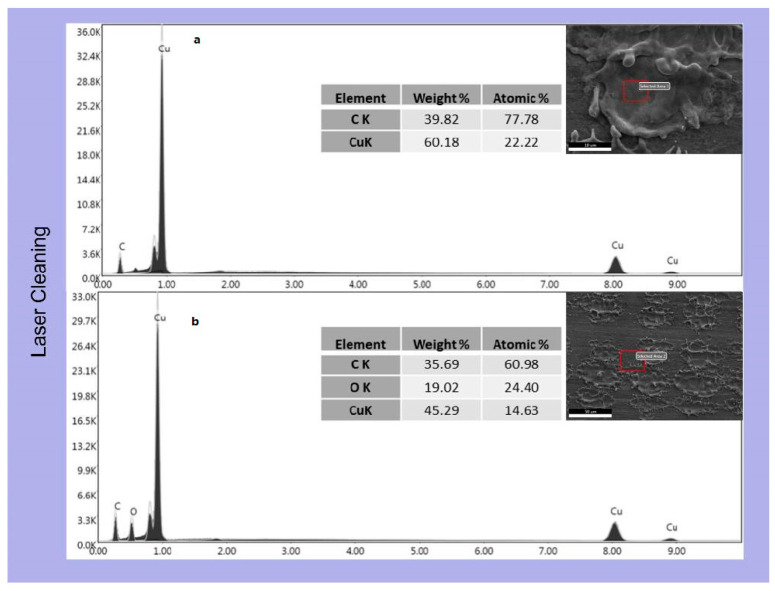
EDX analysis of the EPD coating at 45 V on the laser-cleaned samples; (**a**) inside the dimple and (**b**) on the plain surface.

**Figure 9:**
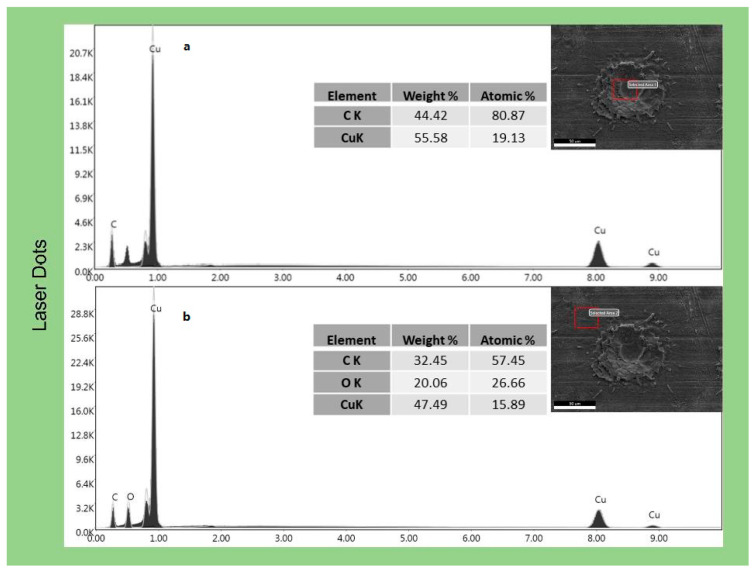
EDX analysis of the EPD coating at 45 V on the laser dot samples; (**a**) inside the dot and (**b**) outside the dot.

**Figure 10:**
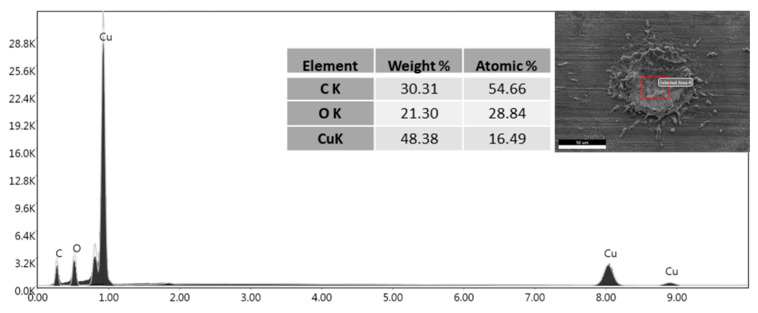
EDX analysis of the EPD coating on the laser dot samplesobtained at 60 V.

EDX analysis (Figure 7) confirmed this behaviour, as the carbon presence on the flat surface produced by pickling and degreasing is notable. EDX analysis of laser-treated samples confirmed how graphene concentrates in the depressions created by the laser both in LC (Figure 8) and in LD (Figure 9). EDX performed outside the craters produced by the interaction with the laser revealed the presence of oxygen due to the oxidation of copper caused by the anodic EPD process. On the other hand, within the cavities, the absence of oxygen peaks indicates the presence of graphene-coated depressions, preventing the oxidation process. At a voltage of 60 V, however, the oxygen peak can also be detected inside the cavity in the LD samples (Figure 10). The high voltages and the charge accumulation on the dots’ edges produce a Faraday cage effect that prevents the graphene from depositing inside the reentrant.

**Figure 11:**
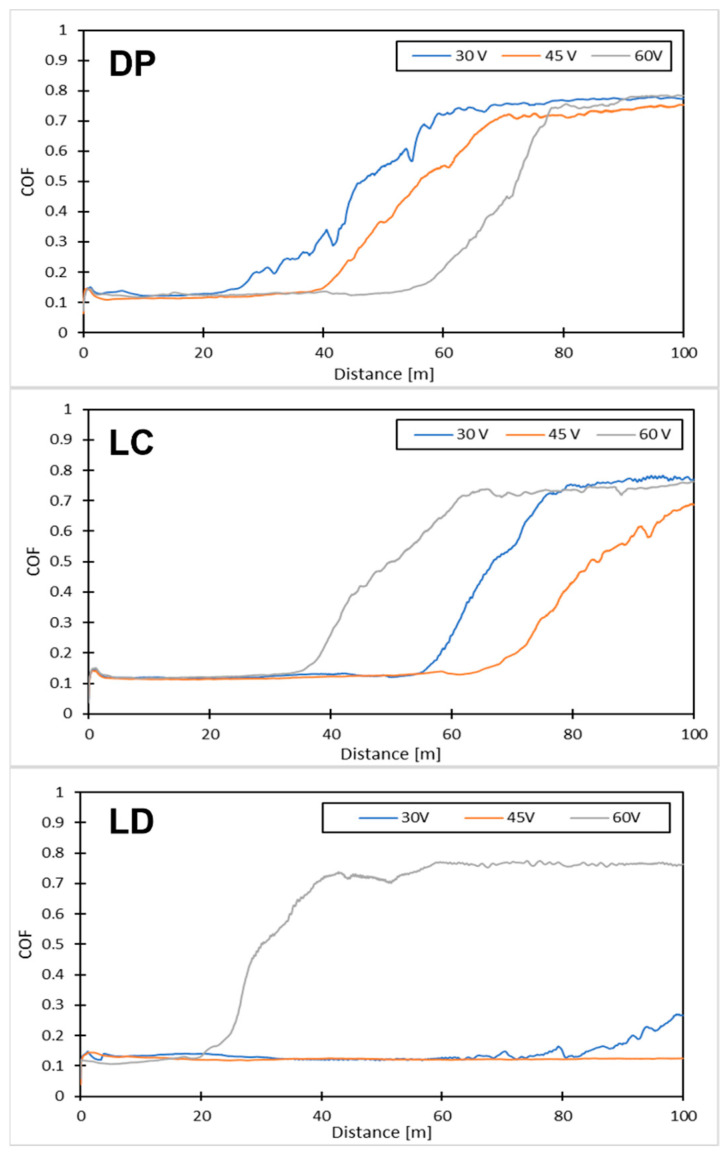
Friction coefficient-sliding distance curves.

**Figure 12:**
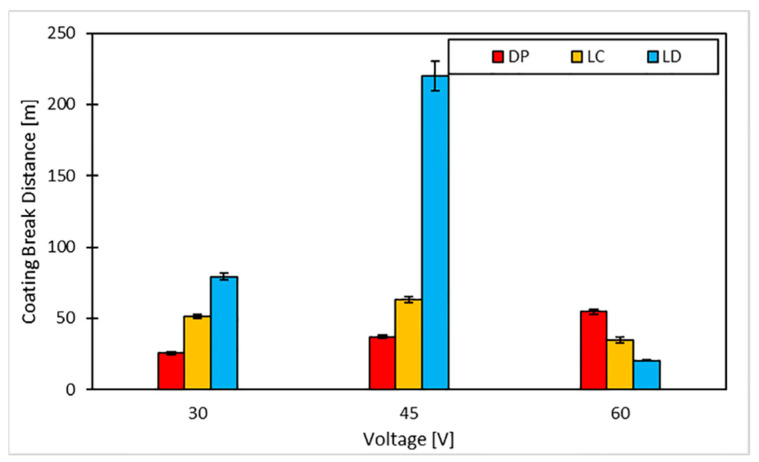
Coating breaking distance achieved for each scenario.

Figure 11 displays the friction coefficient-sliding distance curves from the dry sliding test, and Figure 12 highlights the CBD for each scenario tested. 

**Figure 13:**
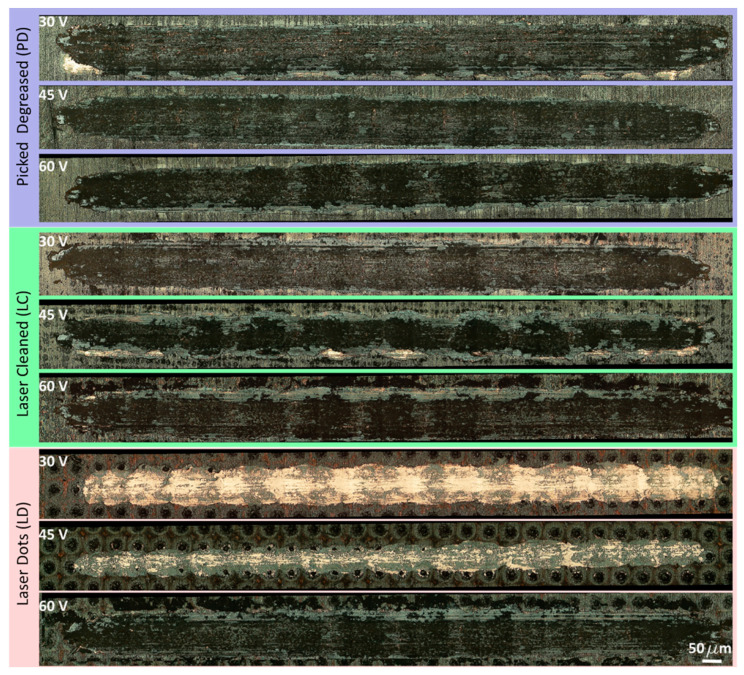
Optical images of the wear tracks.

All of the depositions performed resulted in a COF plateau of about 0.12, indicating the effectiveness of the surface treatment while differing in CBD. The DP surfaces show a CBD that increases linearly with the deposition potential. The levelled surface produced causes the charges to distribute evenly, creating a continuous film. In accordance with [53], the increase in potential entails a higher amount of deposited graphene; since the surface area involved is extensive, the effect of the potential is attenuated, producing only a slight increase in the amount of deposited graphene and, as a result, the coating lifetime. Laser-machined surfaces, on the other hand, exhibit maximum CBD at the intermediate voltage. This behaviour is apparent for the LD surfaces. Above a specific voltage value, the accumulation of charges on the edges of the depressions created by the laser triggers a Faraday cage effect that hinders the deposition of graphene within them and favours the oxidation of the metal, as demonstrated by our EDX analysis. This phenomenon is dampened for the LC, as the surface irregularities are less deep than those created with the LD. Figure 13 reports the wear tracks produced by the wear tests on the different samples. In brief, as shown in Table 7, the low and intermediate voltagesyield better LD performance than the other treatments, considering both wear track depth and width. However, regarding the higher voltages, the scenario is reversed, with the DP samples showing better wear behaviour. Overall, the best coating performance is achieved with an LD treatment and a deposition carried out at a voltage of 45 V.

### 3.3. ANOVA Results

Table 8 reports the ANOVA results in terms of F-Values and *p*-Values. The analysis indicates that the treatment is statistically significant for all of the response variables, while the potential is significant only for CBD. The interaction between treatment and voltage is significant for all the responses variables. 

**Figure 14:**
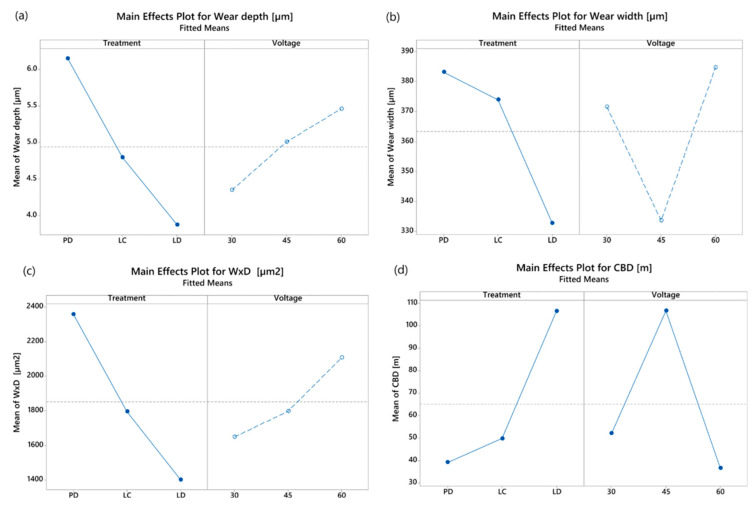
Main effect plots for (**a**) wear depth, (**b**) wear width, (**c**) w × d, and (**d**) CBD; not statistically significant plots are in dashed lines.

Figure 14 shows the main effect plots for wear depth, width, volume (w × d), and CBD; the not statistically significant plots are in dashed lines. 

The LD sample achieves minimal width, depth and w × d products (an index of wear volume: graphene tends to remain inside the dots, acting as a lubricant reservoir. The same applies to CBD, which reaches the highest values with such treatment. 

Concerning the effect of voltage, this is not significant for depth, width, and volume; on the other hand, it is worth emphasising how its interaction is significant. 

**Figure 15:**
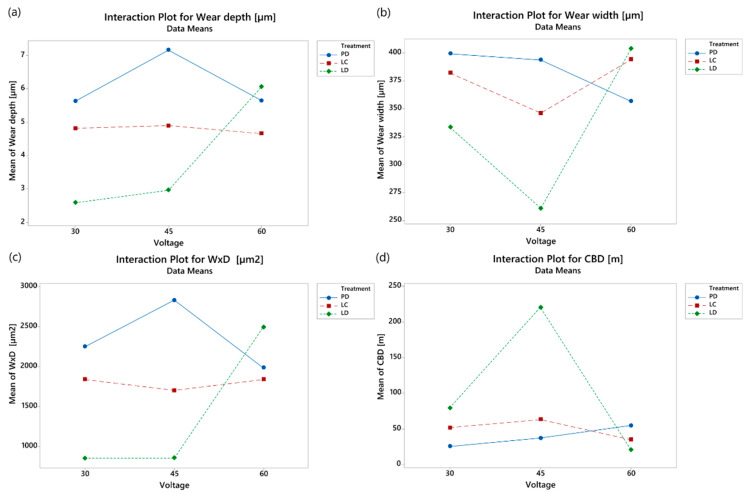
Interaction plots: (**a**) wear depth, (**b**) wear width, (**c**) w × d, and (**d**) CBD; not statistically significant interactions are masked or not reported.

Figure 15 shows that DP varies little with voltage, with a maximum of 45 V, and similar considerations apply to LC; the interaction is significant due to the behaviour of LD; the depth remains constant from 30 to 45 V, and the width (w) presents a minimum at the intermediate voltage. This behaviour is very evident in the case of CBD. DP and LC show an almost constant value as voltage increases, while LD displays a large maximum at 45 V. The homogenous surfaces are not affected by voltage. On the contrary, surfaces with severe irregularities show a high sensitivity to this factor. Under these conditions, the best compromise is the graphene deposition within the dots with a low surface roughness.

## 4. Conclusions

This article presented an analysis of different types of surface preparation treatments on copper substrates, investigating how these affect the deposition of graphene by electrophoresis as a function of the applied process potential. Preliminary analyses have shown that the excessively smooth substrates and highly rough surfaces typical of electropolished and sandblasted samples, respectively, are not conducive to graphene film creation. In fact, although all deposits present a similar coefficient of friction (close to about 0.1), they exhibit a very low CBD.

For the laser and degreasing and pickling treatments, the influence on the electrophoretic process was evaluated, and we also investigated how the potential varies the deposition. A larger dot size favours the adhesion of graphene, which acts as a lubricant reservoir during sliding. However, due to the more pronounced depressions, the process is more sensitive to the applied potential due to the Faraday Cage effect. In general, the lower the roughness of the sample, the lower the sensitivity of the process to this factor, as demonstrated by ANOVA.

## Figures and Tables

**Table 1:** Electropolishing bath composition.

Component	Weight %
Distilled Water	45
Ethanol	25
Phosphoric Acid	25
Isopropanol	5

**Table 2:** Laser system characteristics.

Characteristic	Symbol	Value	Unit
Wavelength	λ	1064	nm
Nominal average power	Pa	30	W
Pulse frequency	F	30÷80	kHz
Pulse duration	D	50	ns
Maximum pulse energy (at F = 30 kHz)	Ep	1	mJ
Maximum pulse power (at F = 30 kHz)	Pp	20	kW
Scanning speed	Ss	*up to* 5000	mm/s
Mode	TEM	00	-
	M^2^	1.2 ÷ 1.5	-
Focused spot diameter (with 160 mm flat field lens)	ds	≈80	μm
Maximum power consumption	-	120	W

**Table 3:** Laser parameters.

Treatment	P [%]	F [kHz]	Ss [mm/s]	Hd [mm]	R
Laser Cleaning	60	30	1000	0.06	1
Laser Dots	100	30	4300	0.16	20

**Table 4:** Graphene features.

Features	Content	Unit
Preparation method	Physical exfoliation	-
Flake Diameter	1–3	μm
Thickness	3–5	nm
Viscosity	1–3	Pa·s
Dispersant	0.5	wt.%
Water	94.5	wt.%
Density	0.06–0.09	g/mL

**Table 5:** Control factors and levels.

Control Factors		Levels		Units
	Low (−1)	Medium (0)	High (+1)	
Treatment	Degreasingand Pickling (DP)	Laser Cleaning (LC)	Laser Dots (LD)	-
Voltage	30	45	60	V

**Table 6:** Roughness values after pretreatments.

	Ra [µm]		Rz [µm]	
	Mean	St. Dev.	Mean	St. Dev.
SB	1.220	0.036	8.489	0.318
EP	0.158	0.010	1.982	0.111
DP	0.223	0.035	3.573	0.669
LC	0.391	0.016	3.689	0.159
LD	0.669	0.200	5.831	1.313

**Table 7:** Wear results on the different samples.

		Wear Depth(d [µm])		Wear Width(w [µm])		w × d [µm^2^]	
Treatment	Voltage	Mean	St. Dev.	Mean	St. Dev.	Mean	St. Dev.
DP	30	5.63	0.1155	399.33	21.3620	2251.20	164.6322
	45	7.17	0.7371	393.67	27.5379	2831.83	472.0609
	60	5.65	0.9677	356.67	48.2113	1985.25	96.2130
LC	30	4.82	0.3253	382.00	3.6056	1840.53	137.9428
	45	4.90	0.4359	346.00	27.5136	1702.83	284.9431
	60	4.67	0.1528	394.00	6.0000	1839.27	87.6745
LD	30	2.59	1.9355	333.33	69.8665	852.45	109.6773
	45	2.97	0.6506	261.00	65.7951	856.40	911.1488
	60	6.07	0.8718	403.67	52.7289	2494.43	869.9069

**Table 8:** ANOVA results in terms of *p*-values and F-values; the statistically significant value are highligted in bold.

Source	Wear Depth, d [µm]		Wear Width, w [µm]		w × d [µm^2^]		CBD [m]	
	F-Value	*p*-Value	F-Value	*p*-Value	F-Value	*p*-Value	F-Value	*p*-Value
Treatment	9.39	**0.002**	3.59	**0.049**	9.49	**0.002**	208.27	**0.000**
Voltage	2.25	0.134	3.51	0.052	2.25	0.134	214.66	**0.000**
Treatment × Voltage	4.16	**0.015**	3.41	**0.030**	6.36	**0.002**	181.18	**0.000**
R-sq [%]	68.92		60.74		73.11		98.87	

## Data Availability

The data are not publicly available but can be made available upon request.
